# Transgastric ERCP is a Useful Modality for Addressing Biliary Complications in Patients After Orthotopic Liver Transplantation and History of Roux‐en‐Y Gastric Bypass: Case Report and Proposed Treatment Algorithm

**DOI:** 10.1002/ccr3.71680

**Published:** 2025-12-17

**Authors:** Nathanael Haynes, William Archie, Maria Baimas‐George, Katheryn Peterson, Vincent Casingal, David Levi, Lon Eskind, Jose Soto, Dionisios Vrochides

**Affiliations:** ^1^ Carolinas Medical Center, Atrium Health Charlotte North Carolina USA

**Keywords:** gastroenterology/hepatology, surgery, transplantation

## Abstract

Biliary complications after orthotopic liver transplantation (OLTx) have a high incidence with inherent risks. Given the rise of obesity and subsequent bariatric surgery, there are new challenges for management, particularly in the setting of bypass anatomy, which is not well described. Trans‐gastric remnant endoscopic retrograde cholangiopancreatography (TG‐ERCP) is a novel technique that could evolve into a primary tool for the diagnosis and treatment of biliary complications after OLTx. A 35‐year‐old female with a history of Roux‐en‐Y gastric bypass (RYGB), decompensated alcoholic cirrhosis, and a MELD of 40 underwent standard OLTx. The postoperative course was complicated by rising liver function tests and common bile duct (CBD) dilatation without graft biliary tree dilation. Due to her bypass anatomy, TG‐ERCP was used to diagnose a biliary stricture which was treated with CBD stenting. A gastric remnant gastrostomy tube (G‐tube) was placed as easy access for all interval ERCP interventions until stricture resolution. As metabolic dysfunction‐associated steatotic liver disease becomes a larger transplant indication, centers will undoubtedly encounter more recipients with RYGB anatomy. When compared to alternative options, TG‐ERCP should be the primary tool for the diagnosis and treatment of postoperative biliary complications, given its high success rate with fewer complications and graft vascular injuries. Future cohort‐based studies are necessary to validate this approach and the proposed treatment algorithm.

## Key Clinical Message

TG‐ERCP serves as an advantageous and acute alternative to other forms of biliary complication management after OLTx in patients with Roux‐en‐Y gastric bypass anatomy, providing a unique means for long‐term enteral access and nutrition intervention.

## Graphical abstract

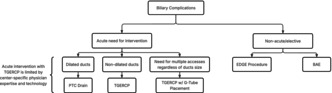

## 1 Introduction

The current and ever‐worsening obesity epidemic has not only accrued a global economic burden of over $1.7 trillion dollars, but has served as the instigating pathology for a wide spectrum of obesity‐related diseases including diabetes, metabolic and cardiovascular diseases, and cancers [1, 2, 3]. As rates of obesity continue to increase in prevalence, healthcare providers have sought to find successful medical and surgical treatment options. Bariatric surgery has emerged as one such effective solution, offering durable and substantial weight loss concomitant with reduction of obesity‐related diseases [1, 4]. Among its range of restrictive and malabsorptive techniques, the Roux‐en‐Y gastric bypass (RYGB) is one of the most performed, given its sustained and superior short and long‐term outcomes [2, 5].

Given this rise in obesity, metabolic dysfunction‐associated steatotic liver disease (MASLD) has become the most common chronic liver disease in developed countries, with projections of MASLD‐related liver transplant waitlist additions to double over the next 10 years [6]. As MASLD becomes a proportionally larger transplant indication, centers will undoubtedly encounter RYGB anatomy. Given the incidence of postoperative biliary complications of up to 30%, this problematic bypass anatomy will make the traditional, transoral endoscopic retrograde cholangiopancreatography (ERCP) unfeasible [7, 8]. As such, biliary interrogation requires a more creative and invasive approach, such as percutaneous transhepatic cholangiography (PTC) drain placement, balloon‐assisted enteroscopy (BAE), or endoscopic ultrasound‐directed trans‐gastric ERCP (EDGE) [9]. These methods are subject to limitations, including highly specialized endoscopists, operative timing, postoperative complication rates, risk of graft vascular injury (PTC), healing time for the lumen‐apposing metal stent (EDGE), or a failure to cannulate the major papilla (BAE) [10, 11, 12]. An alternative tactic that may minimize these constraints is the laparoscopy‐assisted endoscopic retrograde cholangiopancreatography (LA‐ERCP), also known as trans‐gastric ERCP (TG‐ERCP).

TG‐ERCP requires a skilled endoscopist and general surgeon, involving access to the peritoneal cavity through a hole in the gastric remnant to allow endoscopic cannulation with ERCP [9, 13, 14]. With higher rates of success, few complications, and an easily reproducible technique in comparison to EDGE, BAE, and EGHAC, TG‐ERCP offers a persuasive argument as a solution for the management of biliary complications in the complex liver transplantation patient with RYGB anatomy [9, 10, 13, 15]. Currently, no literature describes such a case, nor a treatment algorithm to guide and standardize the approach. As such, this study seeks to begin this discussion with a patient presentation and literature review of biliary complications after liver transplantation in patients with RYGB anatomy to aid in the management of patients who present similarly.

## 2 Materials and Methods

Both institutional review board approval and patient informed consent were obtained, under the same terms outlined in Wiley's standard consent form. Chart abstraction was used for case report details. A literature review was conducted using the PubMed search engine for TG‐ERCP after transplantation.

## 3 Case History/Examination

A 35‐year‐old female with decompensated alcoholic cirrhosis, complicated by hepatic encephalopathy, acute kidney injury, anasarca, and a MELD of 40 was evaluated for orthotopic liver transplantation (OLTx) in the medical intensive care unit in May of 2023. Of note, she had a prior history of RYGB for a BMI of 42.80 in 2007. At the time of consultation, she had achieved a weight loss of approximately −2% (BMI 43.63). An ABO compatible deceased donor was found, and the patient underwent standard OLTx with duct‐to‐duct anastomosis (5–0 PDS, interrupted suture approach) with spatulation to correct for a duct size mismatch (recipient duct: 8 mm, donor duct: 5 mm). There were no unexpected intraoperative findings, and the surgical procedure was completed without complications.

**FIGURE 1:**
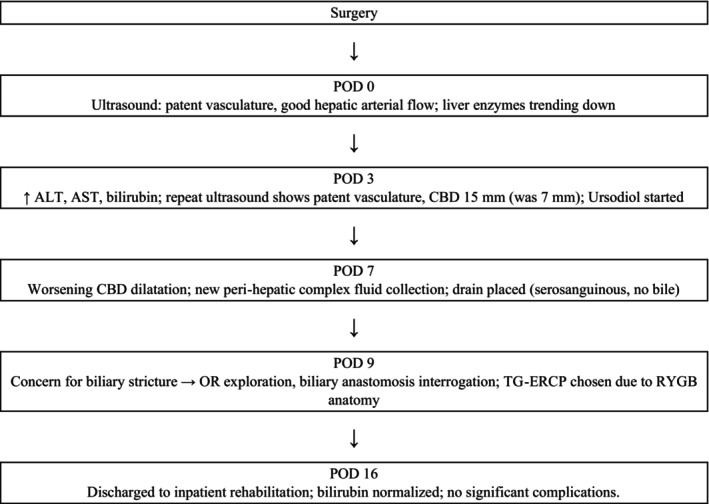
Timeline of clinical events.

Immediately after surgery, ultrasound demonstrated patent vasculature with good hepatic arterial flow (Figure 1). The patient's liver enzymes initially began to downtrend until postoperative day (POD) three when there was an elevation in alanine transaminase (ALT), aspartate aminotransferase (AST), and bilirubin (from 28.9 mg/dL preoperatively to 23.5 mg/dL on POD2 to 29.7 mg/dL on POD3). Repeat ultrasound was reassuring with patent vasculature; however, a distended common bile duct (CBD) to 15 mm was visualized (from 7 mm). The patient was started on ursodiol; however, her bilirubin remained elevated over the next 6 days. Ultrasound on POD7 revealed worsening CBD dilatation with a new peri‐hepatic complex fluid collection. The fluid collection lay behind the right lobe and extended into the pericolic gutter, measuring 11.7 × 8.0 × 7.0 cm. An ultrasound‐guided drain was placed with return of serosanguinous fluid and no evidence of bile; thus, postoperative biloma was effectively ruled out.

## 4 Differential Diagnosis, Investigations and Treatment

On POD9, given this amalgamation of findings, there was ongoing concern for biliary stricture. The decision was made to proceed to the operating room for exploration and interrogation of the biliary anastomosis. There were extensive newly formed inflammatory adhesions from the omentum to the upper anterior abdominal wall, as well as multiple older adhesions from the patient's previous gastric bypass. They were all carefully lysed. The liver appeared cholestatic. No bilomas were seen. The hepatic artery was evaluated by intraoperative ultrasound and was wide open with excellent flow. The hepatic neo‐triad was inspected and noted to appear significantly inflamed and friable, thus making further dissection and possible surgical repair unsafe. Thus, the decision was made to conduct TG‐ERCP, given the patient's RYGB anatomy.

**FIGURE 2:**
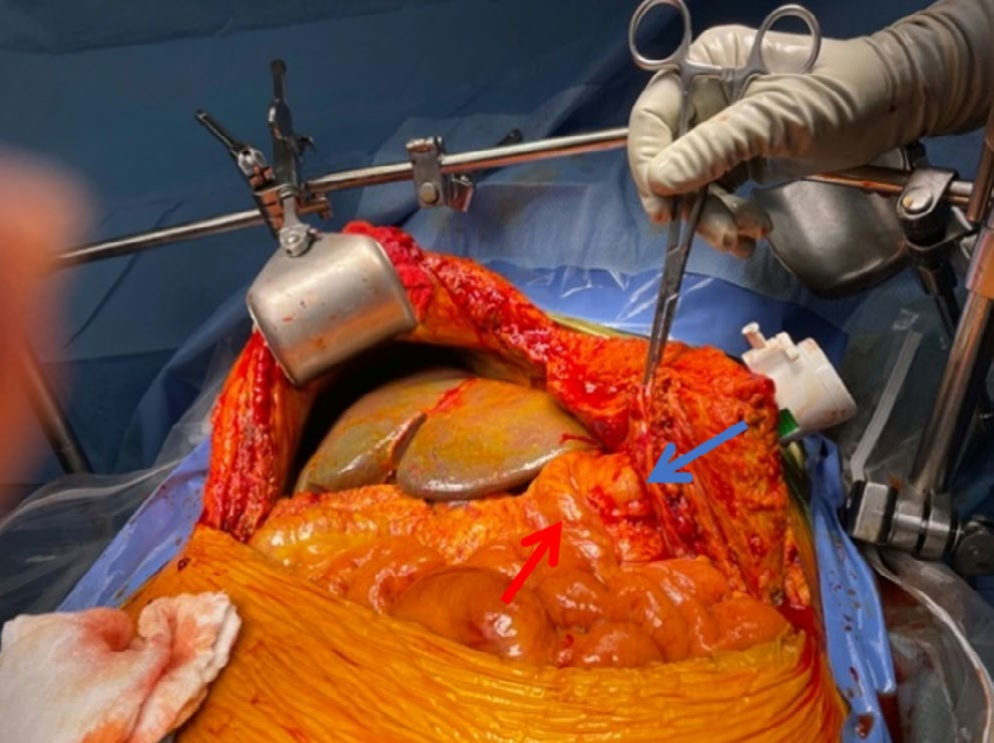
Reopening of the recent transplant incision to access the gastric remnant, positioned away from the recently reconstructed portal triad. A 15 mm trocar is inserted into the remnant stomach (blue arrow), lateral to the Roux limb (red arrow), to optimize endoscope placement. The gastric remnant is secured to the abdominal wall. Red arrow, roux limb. Blue arrow, trocar entering remnant stomach.

**FIGURE 3:**
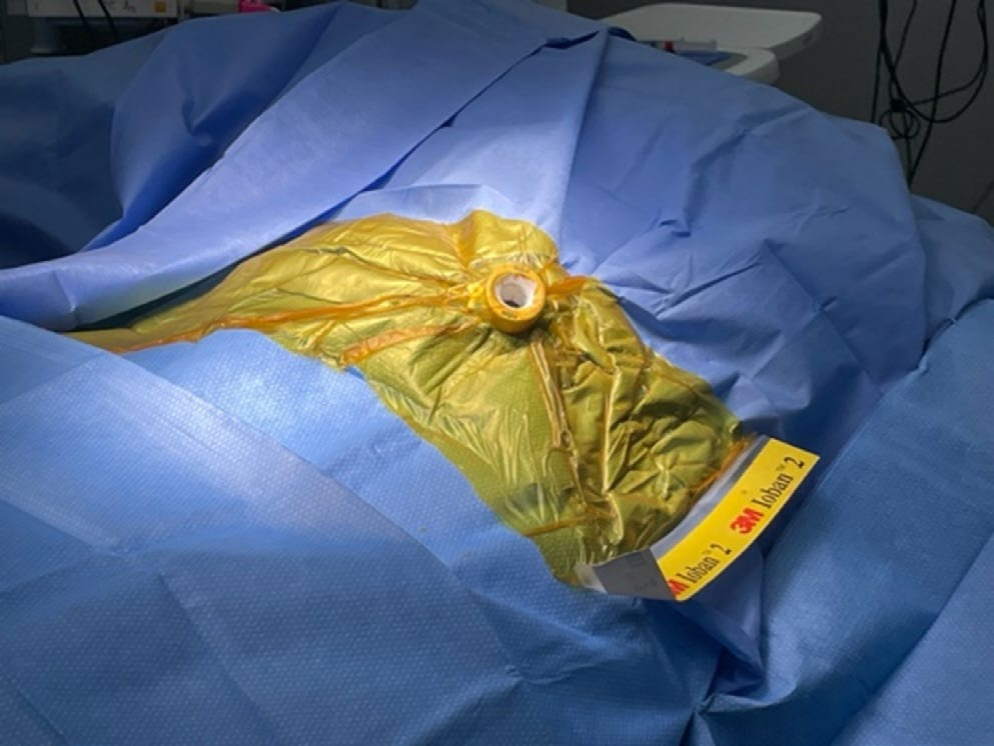
After placement of the 15 mm trocar, the incision is closed, and the sterile field is completely redraped (pictured), allowing the endoscopist controlled access through the trocar, as shown. Upon completion of the endoscopic retrograde cholangiopancreatography (ERCP), the trocar and all overlying drapes are removed en bloc to preserve the sterility of the surgical field. A gastrostomy tube is then inserted through the established trocar site.

**FIGURE 4:**
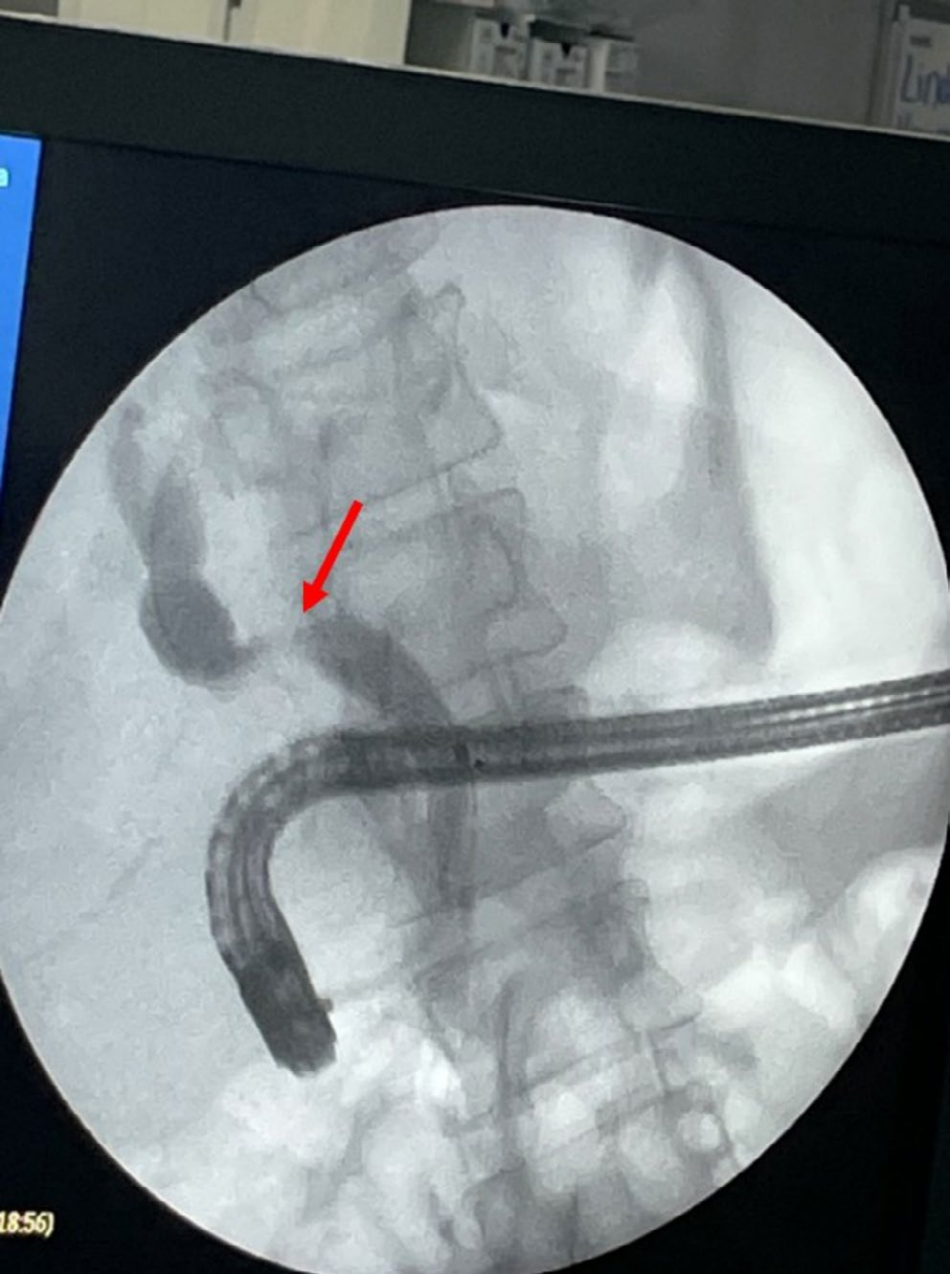
A short, tight anastomotic stricture (red arrow) was identified and a 10 cm 7fr plastic stent was placed across it. Red arrow, anastomotic stricture.

**FIGURE 5:**
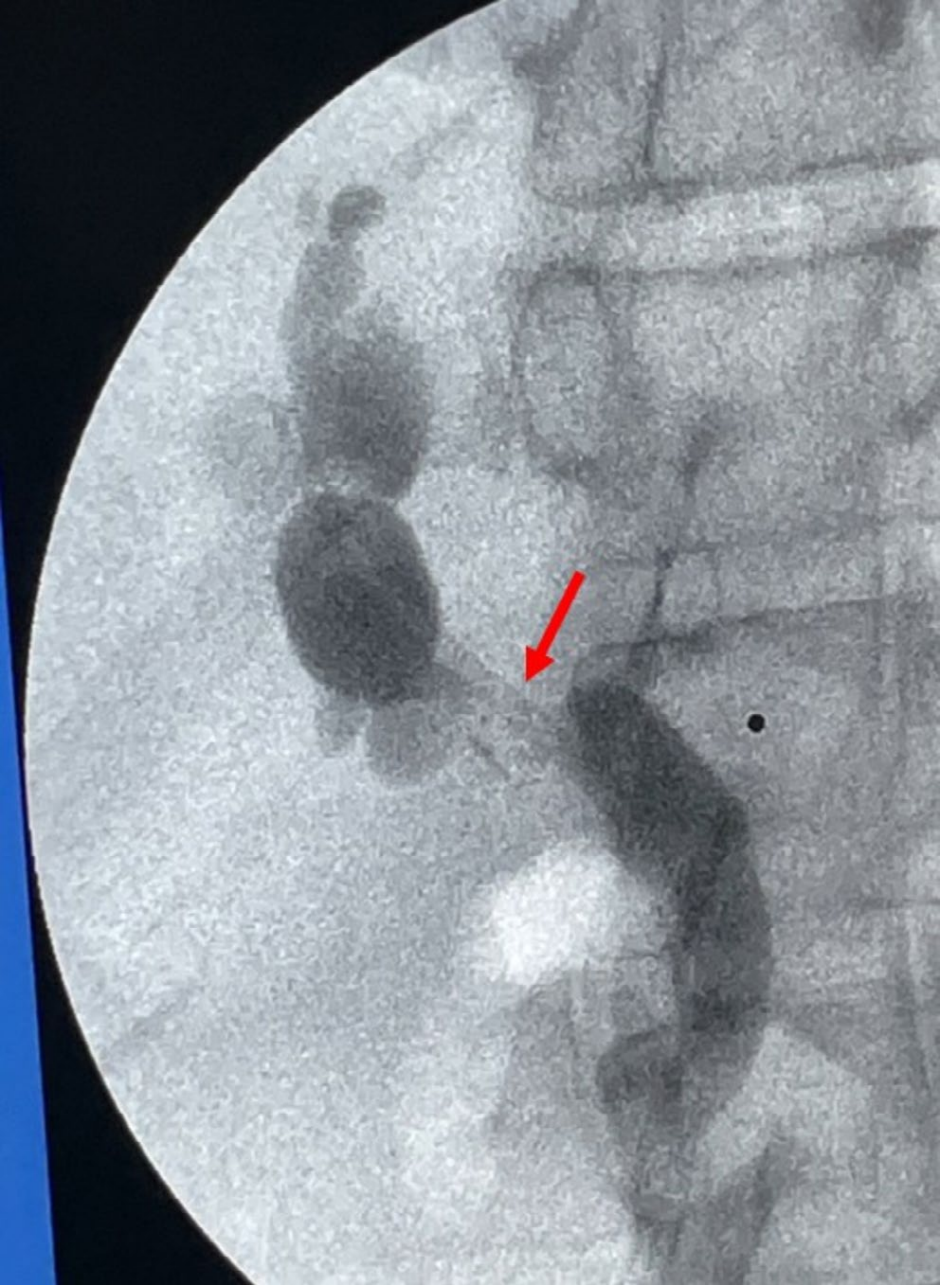
Repeat image of anastomotic stricture (red arrow) after placement of 10 cm 7fr plastic stent. Red arrow, anastomotic stricture.

To proceed with TG‐ERCP, the roux limb and gastric remnant were both identified and dissected. The gastric remnant was mobilized, and an abdominal wall incision was made in the upper left quadrant above the left extension of the Mercedes incision. A gastrotomy was made, and a 15 mm trocar was placed through it into the gastric lumen, as previously described (Figure 2) [14]. The anterior wall of the remnant was then stammed to the abdominal wall with a 3–0 barbed suture. The abdominal cavity was temporarily closed, and overlying drapes were placed, allowing access solely to the 15 mm trocar (Figure 3). The advanced endoscopist then cannulated the trocar with the side‐viewing endoscope, and an ERCP was performed in the usual fashion (Figure 4). A single biliary stricture was identified at the anastomosis, and a 10 cm, 7‐French plastic stent was placed across (Figure 5). No balloon dilation of the anastomosis was performed due to the recentness of the anastomosis. The trocar and the overlying drapes were removed, and a 24‐French gastrostomy tube (G‐tube) was placed through the gastrotomy for future biliary access and enteral nutrition, if deemed necessary. Liver biopsy was performed that demonstrated marked canalicular cholestasis and bile ductular proliferation consistent with the biliary obstruction. There was no evidence of rejection.

## 5 Conclusions and Results

Postoperatively, the patient's bilirubin and liver enzymes began to decline. She had no other significant postoperative complications. After 7 days in the hospital, she was discharged to inpatient rehabilitation with normalizing bilirubin. She was scheduled to undergo a repeat TG‐ERCP for stent removal, this time in the endoscopy suite, given the gastric remnant access via the G‐tube negates the need for a repeat operative exposure.

Use of TG‐ERCP for biliary complications after OLTx in patients with RYGB shows promise as a successful strategy with distinct advantages. It serves as an acute alternative to the EDGE procedure, with higher success rates than BAE and fewer complications and contraindications than PTC drainage, while also providing long‐term enteral access for future intervention and nutrition. Future cohort‐based studies are needed to evaluate TG‐ERCP within the transplant population, as well as validate the proposed algorithmic treatment pathway for biliary complications after OLTx in patients with RYGB anatomy, as well as its potential application to patients with hepaticojejunostomies.

## 6 Discussion

The incidence of biliary complications in liver transplant patients is not diminutive and has increased recently, likely due to the extension of organ criteria for donor use [16]. With postoperative biliary complications reaching as high as 25%, appropriate and timely management is paramount, but complicated by a parallel rise in bariatric surgery and subsequent bypass anatomy [15, 17]. As such, alternate techniques need to be considered and evaluated. This case study presents one such approach, TG‐ERCP, in a 35‐year‐old female with a postoperative biliary stricture after OLTx.

In this case presentation, the use of TG‐ERCP for management of a postoperative biliary stricture was successful as evidenced by down trending post‐procedural bilirubin and liver function enzymes and resolution of the CBD dilation. There were no postoperative complications, and the patient was discharged to inpatient rehabilitation after 7 days. The procedure not only treated the stricture but also allowed for enteral tube placement through the gastrotomy. This strategy gives the endoscopist access outside of the operating room for easy stent removal and/or future, more advanced management if the initial plastic stent fails, such as long‐term intubation with a removable metal stent.

Our center has performed over 1500 liver transplants, of which 23 patients had previous RYGB. Of this cohort, four patients reported biliary complications within the first year: CBD stricture (*n* = 2), biliary leak (*n* = 2). Three were treated with a combination of PTC drain placement and operative revision. The fourth was treated with TG‐ERCP and is described in this case report. This is the first and only instance of treating a biliary stricture with TG‐ERCP at our institution.

**FIGURE 6:**
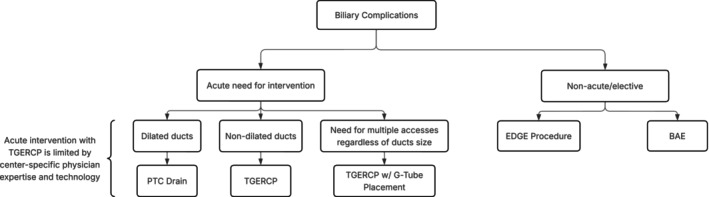
Proposed algorithm for diagnosis and management of postoperative biliary complications after orthotopic liver transplantation in patients with Roux‐en‐Y gastric bypass anatomy. BAE, balloon‐assisted enteroscopy, EDGE, endoscopic ultrasound directed ERCP, G‐tube, gastrostomy tube, PTC drain, percutaneous transhepatic cholangiography drain, TGERCP, transgastric remnant endoscopic retrograde cholangiopancreatography.

Our single‐center favorable experience with TG‐ERCP is consistent with the current literature around non‐OLTx patients, which notes high success rates of 90%–100% for management of biliary postoperative pathology [9, 13]. Unfortunately, in the setting of OLTx and RYGB, there is limited literature evaluating biliary interrogation techniques with this method. Given our experience and literature review, we suggest TG‐ERCP is a viable first‐line approach under certain circumstances (Figure 6). TG‐ERCP uses a single port through remnant gastrotomy, which allows for minimal dissection, reducing surgical trauma and avoiding the recently reconstructed portal triad [18]. It eliminates the higher risk of graft vascular injury by PTC drain placement, particularly in patients with nondilated bile ducts, as well as PTC contraindications of ascites and compromised coagulation [12, 19]. It overcomes BAE's lower rate of successful biliary complication management, largely confounded by the challenge of standard cannulation of the intact papilla in RYGB patients [9, 20]. Further, TG‐ERCP can be performed acutely for potentially life‐threatening biliary complications in the setting of a surgically inaccessible hilum, unlike the two‐staged EDGE procedure that requires approximately 2 weeks of transluminal tract maturation in most instances [21]. In addition, utilization of the TG‐ERCP placed G‐tube is a solution to future biliary access needs and the requirement for postoperative stent removal. Furthermore, it has the capability to address malnourishment by enteral supplemental nutrition. Finally, TG‐ERCP appears to be a simple procedure in single‐center experiences, after gastric remnant access is established, given the widespread modern adoption of ERCP. In fact, this has the potential to allow for minimization of center‐to‐center variability by lowering both expertise and technological performance thresholds [10, 13, 18].

TG‐ERCP is not without drawbacks (Table 1). An additional operation is required to facilitate endoscopic access and presents a longer procedure duration. However, this operation can be done laparoscopically or robotically depending on surgeon experience and expertise. In our case, our original goal in returning to the operating room was exploration with possible biliary reconstruction prior to the decision to perform TG‐ERCP. Regardless, we would not recommend prophylactic mobilization of the remnant stomach or G‐tube placement due to the risk of bleeding from the spleen and short gastrics in patients with portal hypertension. Therefore, for patients requiring acute or emergent intervention with either contraindications to PTC drain placement or concurrent need for supplemental enteral nutrition, TG‐ERCP should be first‐line intervention.

**TABLE 1:** Comparison of available techniques for the management of biliary stricture in the setting of Roux‐en‐Y gastric bypass anatomy.

Procedure	TG‐ERCP	EDGE	EUS‐BD	BAE	PTC drain
Availability	Simple procedure at our center that addresses acuity of case presentation; requires OR, surgical and endoscopic teams	Limited to advanced centers; requires EUS/ERCP expertise	Requires OR, surgical and endoscopic teams	Widely available; no surgical support needed; requires a skilled endoscopist	Widely available; interventional radiology
Invasiveness	Invasive (surgical interrogation of gastric remnant)	Minimally invasive (endoscopic)	Minimally invasive (endoscopic)	Minimally invasive (endoscopic)	Invasive (percutaneous, external drain)
Procedure duration	Longer in Non‐OLTx (144.8 min)	Longer (97.6 min)	Shorter (55 min)	Longer (95 min)	Shorter (43.5 min)
Technical success	High in Non‐OLTx (90–100%)	High (98.6%)	High (93.4%)	Lower (75.8%), due to failure to cannulate the papilla	High (92.3%; as low as 68% in patients with nondilated ducts)
Clinical success	High in Non‐OLTx (90–100%)	High (97.9%)	High (83.4%–88.0%)	Lower (65%–75%)	Moderate (69%–88.6%)
Adverse events	Bleeding, infection, reintervention	Weight loss, persistent fistula	Bile leak, bleeding, infection	Perforation, pancreatitis, bleeding	Pain, infection, dislodgement, reintervention
References	[9, 13, 18]	[22, 23]	[22, 24, 25]	[25]	[22, 26, 27]

Abbreviations: BAE, balloon‐assisted enteroscopy; EDGE, endoscopic ultrasound directed ERCP; EUS‐BD, endoscopic ultrasound guided biliary drainage; PTC drain, percutaneous transhepatic cholangiography drain; TGERCP, transgastric remnant endoscopic retrograde cholangiopancreatography.

While promising, our conclusions require validation, ideally with prospective multicenter studies to evaluate the impact of TG‐ERCP in the liver transplant population on outcomes, cost, and resource utilization.

## Author Contributions

**Nathanael Haynes:** conceptualization, data curation, formal analysis, investigation, methodology, project administration, writing – original draft, writing – review and editing. **William Archie:** formal analysis, project administration, writing – original draft, writing – review and editing. **Maria Baimas‐George:** formal analysis, writing – original draft, writing – review and editing. **Katheryn Peterson:** data curation, writing – review and editing. **Vincent Casingal:** conceptualization, writing – review and editing. **David Levi:** conceptualization, writing – review and editing. **Lon Eskind:** conceptualization, writing – review and editing. **Jose Soto:** conceptualization, writing – review and editing. **Dionisios Vrochides:** conceptualization, formal analysis, methodology, project administration, resources, supervision, writing – original draft, writing – review and editing.

## Funding

The authors have nothing to report.

## Disclosure

The authors have nothing to report.

## Ethics Statement

This study was approved by the Atrium Health—Wake Forest Baptist Institutional Review Board. The Atrium Health—Wake Forest Baptist Institutional Review Board was consulted for any ethical considerations.

## Consent

Written informed consent was obtained from the patient required to publish this report. Written patient consent was obtained in accordance with Clinical Case Report's patient consent policy. This study was conducted in accordance with institutional guidelines and best practices for retrospective research, including appropriate data handling and confidentiality.

## Conflicts of Interest

The authors declare no conflicts of interest.

## Data Availability

Data sharing not applicable to this article as no datasets were generated or analyzed during the current study.
